# The benefit of dupilumab as a postoperative short‐term adjuvant therapy for chronic rhinosinusitis with nasal polyps: A preliminary study

**DOI:** 10.1002/lio2.1296

**Published:** 2024-07-03

**Authors:** Pei‐Wen Wu, Chi‐Che Huang, Po‐Hung Chang, Ta‐Jen Lee, Chien‐Chia Huang

**Affiliations:** ^1^ Division of Rhinology, Department of Otolaryngology Chang Gung Memorial Hospital and Chang Gung University Taoyuan Taiwan; ^2^ School of Medicine Chang Gung University Taoyuan Taiwan

**Keywords:** adjuvant therapy, biologic, chronic rhinosinusitis with nasal polyp, dupilumab, functional endoscopic sinus surgery

## Abstract

**Introduction:**

Recent evidence recommends the use of biologics in patients with severe uncontrolled type 2 chronic rhinosinusitis with nasal polyp (CRSwNP) owing to its propensity for recurrence after functional endoscopic sinus surgery (FESS). Among the type 2 biologics used for the treatment of nasal polyps, dupilumab (Dupi, anti‐IL‐4) exhibited superior efficacy and safety in indirect comparison studies.

**Objective:**

This study aimed to evaluate the objective and subjective outcomes of patients with CRSwNP treated with and without adjuvant Dupi therapy after FESS.

**Methods:**

Adult patients with type 2 CRSwNP who underwent FESS with adjuvant Dupi after surgery were enrolled. A matched control group without adjuvant Dupi therapy were recruited during the same period. All patients underwent nasal endoscopy and completed the sinonasal outcome test‐22 questionnaire evaluations at baseline and 3 months after surgery.

**Results:**

A total of 10 patients who received postoperative adjuvant therapy with Dupi and 20 patients who underwent surgery only were included. Patients with add‐on Dupi therapy had significantly higher eosinophil cationic protein levels in the serum, eosinophil counts in peripheral blood, prevalence of asthma, and nasal polyp score at baseline. Both treatments were effective in reducing the patient's symptoms by SNOT‐22 at 3 months postoperatively. However, patients with adjuvant Dupi therapy exhibited significantly better endoscopic scores than those with surgery only (*p* = .022).

**Conclusion:**

Surgery plays an important role in treating patients with CRSwNP, and adjuvant Dupi use may facilitate objective mucosal recovery postoperatively.

**Level of Evidence:**

4.

## Graphical abstract

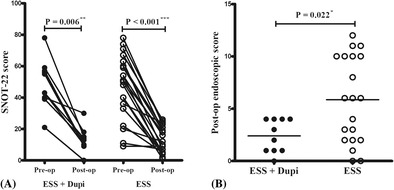

## 1 INTRODUCTION

The prevalence of chronic rhinosinusitis with nasal polyps (CRSwNP) is estimated to be 2.1% to 8.4% in Asia.^1^ Patients with CRSwNP and a type 2 pattern of inflammation are considered as challenging cases in clinical practice. Despite appropriate therapy with oral/topical corticosteroids, regular saline irrigation, and comprehensive sinus surgery (FESS), severe type 2 CRSwNP remains a challenging clinical problem owing to its propensity for recurrence.^1^ The recurrence rate of CRSwNP in southwest China was 21.8% over 8 years post‐surgery and 55.3% in northeast China approximately 34 months post‐surgery.^1^ It is therefore imperative to find a more effective treatment for recalcitrant CRSwNP.

Recent evidence recommends the use of biologics in patients with severe uncontrolled type 2 CRSwNP.^2^ Among the type 2 biologics used for the treatment of nasal polyps, dupilumab (Dupi, anti‐IL‐4) exhibited superior efficacy and safety in indirect comparison studies. However, no study has considered the benefits of biologics as a postoperative short‐term adjuvant therapy for severe type 2 CRSwNP. Thus, this study aimed to evaluate the objective and subjective outcomes of patients with CRSwNP treated with and without adjuvant Dupi therapy after FESS.

After obtaining approval from the Institutional Review Board (IRB number: 202102257A3), adult patients with type 2 CRSwNP who underwent FESS between March 2022 and February 2023 with adjuvant Dupi after surgery were enrolled. The diagnosis of type 2 inflammation was based on the EPOS 2020 definition, with blood eosinophil (EOS) counts ≥250 μ/L or total immunoglobulin E (IgE) level ≥100 IU/mL.^2^ Dupi was administered with a pre‐filled syringe through a subcutaneous injection at a dose of 300 mg. The first injection was administered under medical control, within 1 week after surgery, followed by once every 2 weeks. The total dose used was ranged from 2 to 6 depending on patients' private insurance coverage. The use of biologic in treating CRSwNP is not covered by the national health insurance. The patients have to pay by themselves and the used dosage is depending on their economic status or the personal insurance coverage. The aim of usage of biologic immediately after sinus surgery was to help mucosal recovery in these patients. Antibiotic therapy with Curam 875/125 mg/tab (Amoxicillin + clavulanic acid, SANDOZ GMBH, Aus) for 3 weeks was prescribed after surgery. All patients were instructed to use nasal saline douching and intranasal corticosteroids postoperatively.

A 1:2 matched control group included patients who underwent FESS for type 2 CRSwNP during the same period. The matching variable included age, preoperative Lund‐Mackay computed tomography scores, aeroallergen test results, history of revision surgery, and preoperative sinonasal outcome test‐22 (SNOT‐22) scores.

All patients underwent nasal endoscopy and completed the SNOT‐22 questionnaire at baseline and 3 months after surgery. For consistency, the endoscopic images were reviewed and scored by a rhinologist (CCH) blinded to the diagnostic information. The preoperative nasal polyp score (NPS) was calculated according to the study by Gevaert et al.^3^ and the modified Lund–Kennedy (MLK) endoscopic score was used to grade postoperative sinonasal inflammation.^4^

A total of 10 patients who received postoperative adjuvant therapy with Dupi and 20 patients who underwent surgery only were included in the study. The clinical and laboratory characteristics of patients are shown in Table 1. Patients with add‐on Dupi therapy had significantly higher eosinophil cationic protein (ECP) levels in the serum, EOS counts in peripheral blood, prevalence of asthma, and preoperative NPS. Three months postoperatively, both treatments were effective in reducing the patient's symptoms. The SNOT‐22 score improved from 49.1 ± 15.4 to 12.9 ± 7.7 in patients who underwent surgery plus Dupi therapy (*p* = .006) and from 49.0 ± 20.3 to 12.9 ± 8.4 in patients who underwent only surgery (*p* <.001). There were no significant differences in the proportional changes in the SNOT‐22 scores and smell symptom scores between the two groups. However, patients who received postoperative adjuvant Dupi therapy exhibited significantly better MLK endoscopic scores (2.1 ± 1.4) than those in patients who underwent surgery only (4.8 ± 3.3; *p* = .022).

**TABLE 1:** Clinical and biological characteristics of patients with and without adjuvant dupilumab therapy.

	With dupilumab	Without dupilumab	*p* value^a^
	(*n* = 10)	(*n* = 20)	
Age (years)	43.1 ± 9.4	42.9 ± 10.3	.741
Female: male	3:7	8:12	.592
Dupilumab doses	4 ± 2	0	
Smoking	5 (50%)	5 (25%)	.171
Revised operation	5 (50%)	9 (45%)	.796
Comorbid asthma	5 (50%)	3 (15%)	.041*
Positive Phadiatop	5 (50%)	10 (50%)	1.000
Serum IgE (KU/L)	244.8 ± 165.1	142.3 ± 149.5	.054
Serum ECP (μg/L)	71.1 ± 63.6	35.4 ± 40	.018*
Eosinophil count (%)	9.7 ± 6.1	4.1 ± 3.7	.005**
Absolute eosinophil count (/μL)	660 ± 416.4	267.1 ± 208.2	.003**
Hyposmia/anosmia	9 (90%)	13 (65%)	.144
Nasal polyp score	5.9 ± 1.1	4.8 ± 1.7	.036*
Preoperative CT L‐M score	17.4 ± 3.8	18.3 ± 3.5	.479
Preoperative CT E/M ratios	2.6 ± 0.7	3.7 ± 1.9	.110
Preoperative SNOT‐22 score	49.1 ± 15.4	49.0 ± 20.3	.878

*Notes*: Data are represented as the mean ± stand deviation.

^a^ Mann–Whitney *U* test for continuous variables; *χ* ^2^ test or Fisher exact test for categorical variables.

Abbreviations: n, number; IgE = Immunoglobulin E; ECP, eosinophil cationic protein; CT, computed tomography; L‐M, Lund‐Mackay; E/M, ethmoid/maxillary; SNOT‐22, sinonasal outcome test‐22;

* *p* < .05;

** *p* < .01.

## 2 DISCUSSION

In the current study, patients who received adjuvant Dupi therapy postoperatively had significantly higher ECP levels in the serum, EOS counts in the peripheral blood, and comorbid asthma rate. All these factors may be related to more severe type‐2 inflammation and a higher recurrence rate after surgery. However, compared to patients who did not undergo adjuvant Dupi therapy, less sinonasal inflammation was observed 3 months after surgery, as revealed by the significantly lower MLK endoscopic scores. Pathological analysis in one patient who received surgery plus adjuvant therapy with Dupi 300 mg every 2 weeks for consecutive 12 weeks demonstrated not only decreased EOS infiltration in the submucosa but also increased expression of tight junction (TJ) proteins in the epithelium. Decreased expression of TJ proteins in interleukin (IL)‐13‐treated epithelial cells has been described previously.^5^ Thus, Dupi may have the potential to facilitate barrier restoration in the postoperative recovery phase by inhibiting both IL‐4 and IL‐13 signaling.

In our study, with or without adjuvant Dupi use, all patients experienced significant improvement in the quality of life, and there was no intergroup difference in the change in SNOT‐22 scores. Overall, surgery plays an important role in treating patients with CRSwNP, and adjuvant Dupi use may facilitate postoperative mucosal recovery by inhibiting type‐2 inflammation and restoring barrier function.

## 3 CONCLUSION

Surgery plays an important role in treating patients with CRSwNP, and adjuvant Dupi use may facilitate objective mucosal recovery postoperatively.

## FUNDING INFORMATION

The authors received research grants from the Taiwan National Science and Technology Council (111‐2314‐B‐182‐067‐). The funder had no role in the study design, data collection and analysis, decision to publish, or manuscript preparation.

## CONFLICT OF INTEREST STATEMENT

The authors declare no conflicts of interest.
